# Pharmacist Dispensing of Mifepristone: Evaluation of Knowledge and Support Before and After a Continuing Education Course

**DOI:** 10.3390/pharmacy13050131

**Published:** 2025-09-15

**Authors:** Natalie Morris, Alexa Orosz, M. Antonia Biggs, Sally Rafie, Daniel Grossman

**Affiliations:** 1 Advancing New Standards in Reproductive Health (ANSIRH), Bixby Center for Global Reproductive Health, Department of Obstetrics, Gynecology and Reproductive Sciences, University of California San Francisco, Oakland, CA 94612, USA; antonia.biggs@ucsf.edu (M.A.B.); daniel.grossman@ucsf.edu (D.G.); 2 School of Pharmacy, University of California, San Francisco, CA 94143, USA; alexa.orosz@ucsf.edu; 3 Birth Control Pharmacist, San Diego, CA 92014, USA; sally@birthcontrolpharmacist.com

**Keywords:** mifepristone, medication abortion, pharmacist continuing education, pharmacist dispensing of mifepristone

## Abstract

Medication abortion with mifepristone and misoprostol is a safe and effective method for ending a pregnancy. Pharmacy dispensing of mifepristone was approved by the U.S. Food and Drug Administration in 2023, but educational opportunities for pharmacists were nonexistent. We designed a 1 h continuing education course on medication abortion for pharmacists, which was offered in a live-webinar or recorded-video format over 3 years. It included key medication abortion topics, including medications, patient counseling, relevant policies, and implementing pharmacy dispensing. Using a prepost design, we administered online surveys to participants prior to and after completing the course to assess changes in overall medication abortion knowledge score (six items, Cronbach’s alpha = 0.76) and support for pharmacist dispensing of medication abortion (one Likert-scaled item). During the study period, 279 students and pharmacists took the course, of which 148 completed both the pre- and post-course questionnaires. Adjusted regression analyses demonstrated significant increases in knowledge scores and support for pharmacist dispensing of medication abortion post-course; most thought dispensing mifepristone would be very (21.6%) or somewhat easy (38.5%) to implement, and 75% indicated a willingness to dispense mifepristone if allowed. These findings suggest that video-based education on medication abortion is an effective tool for enhancing pharmacists’ knowledge and support for medication abortion, which could increase access to reproductive health care.

## 1. Introduction

Medication abortion (MA) is a method for terminating an early pregnancy using a combination of two drugs, mifepristone and misoprostol [1]. This method is recognized as safe and effective and is widely utilized by those seeking abortion care [2]. The U.S. Food and Drug Administration (FDA) previously required prescribers to dispense mifepristone to patients in person. However, based on accumulated safety data, in 2021, the FDA announced it would modify the drug’s Risk Evaluation and Mitigation Strategy (REMS) to allow mailing of mifepristone, including by mail-order pharmacies, and in 2023, the agency issued guidance for pharmacies to become certified to dispense mifepristone prescriptions [1].

Despite the policy change to allow pharmacists to dispense mifepristone in the U.S., pharmacy certification has increased slowly, even in states where abortion remains broadly legal [3,4,5]. Given the many barriers to pharmacy certification, including requirements imposed by the FDA, there exists a need to educate pharmacists about the medication and its REMS in order to increase uptake of pharmacy dispensing of MA [6].

In Australia, pharmacists have been able to dispense mifepristone on prescription since the medication was approved in 2012, which has helped to expand access to MA across the country [7]. In 2015, a study evaluating the implementation of this model found that there was a general lack of clinical, ethical, and legal knowledge about medication abortion among Australian pharmacists [8]. This indicates that implementation of such a model still requires pharmacist-specific training to ensure pharmacists are well-equipped to offer patients high-quality MA care.

Regulatory changes regarding reproductive health medications may lead to challenges in disseminating and operationalizing pharmacist dispensing of these medications. A U.S. study found that pharmacies in low-income neighborhoods were more likely to provide incorrect information on emergency contraceptives (EC) to mystery callers posing as adolescents seeking the medication, denying them EC when they were actually eligible [9]. Similarly, another nationwide study found that 40% of pharmacies incorrectly reported an age restriction for EC, limiting access for potential purchasers [10]. Other studies assessing pharmacy dispensing of the EC ulipristal acetate (UPA) found that pharmacists often provide inaccurate information about UPA effectiveness [11,12]. These results highlight the need for continuing pharmacist education to support expanding access to reproductive health medications.

Several studies have demonstrated that pharmacists are willing to dispense mifepristone for MA patients [13]. However, this research also identified a need for training on the topic since abortion care is rarely included in pharmacy training [14]. Pharmacists regularly update their knowledge on new drug therapies as part of their professional duties and could similarly receive education on MA if formal education on it is lacking [14].

Prior to the FDA REMS modification, a clinical trial that trained pharmacists to dispense mifepristone to MA patients found that knowledge and support for a pharmacist-dispensing model increased significantly by the study’s end [15]. Most participating pharmacists (95%) found the in-person training to be somewhat or very sufficient in preparing them to dispense MA medications.

The objective of this study was to assess whether participating in a continuing education module was associated with improvements in knowledge about MA with mifepristone and misoprostol and increased support for pharmacy dispensing of mifepristone.

## 2. Materials and Methods

### 2.1. Study Design

Starting in 2019, a group of pharmacists and clinicians developed an evidence-based online pharmacist continuing education (CE) module [16], aimed at training practicing pharmacists, pharmacy residents, and pharmacy students on dispensing mifepristone on prescription for MA. The course was offered in three sessions: as a recorded video for home study from September 2020 to May 2023 (session 1), a live webinar in June 2023 (session 2), and a video recording of the live webinar for home study thereafter (session 3).

The 1 h course included information about the landscape of abortion in the U.S., access to abortion care, and methods of abortion, and then focused on more details about MA. The course described how clinicians counsel patients about MA, how eligibility is assessed, the medications’ mechanism of action, common side effects and their management, symptoms that may indicate a complication, follow-up care, drug safety, postabortion contraception, other uses of the medications, regulatory issues, and the specific role of the pharmacist in dispensing mifepristone and misoprostol, including counseling. The course was updated in 2023 for the live webinar to include a brief overview of legal changes since the U.S. Supreme Court’s decision in *Dobbs v. Jackson Women’s Health Organization*, as well as changes to the mifepristone REMS and pharmacy certification requirements to dispense.

From September 2020 through February 2024, we invited course participants to complete two short online surveys, one prior to the course (pre-course) and one immediately after taking the course (post-course). The Institutional Review Board of the University of California, San Francisco, determined that this study was exempt from federal policy for the protection of human subjects.

### 2.2. Measures

Our primary and secondary outcome measures included MA knowledge and support for pharmacy dispensing of mifepristone, which were measured pre-course and post-course, as well as perspectives on medication abortion dispensing.

Medication abortion knowledge: Due to the absence of validated measures assessing pharmacists’ knowledge of medication abortion, we developed six MA knowledge items with input from experts, including abortion providers, pharmacists, and researchers [15]. The items were pilot-tested with a sample of pharmacists and revised accordingly. These six items focused on (1) mifepristone and (2) misoprostol mechanisms of action, (3) misoprostol dosage and administration, (4) common side effects, (5) postabortion return to fertility, and (6) FDA gestational duration limits on MA. Answer options were multiple-choice with only one correct answer and an “I don’t know” option. All six items were coded as dichotomous variables: “correctly answered” or “incorrectly answered.” Responses of “I don’t know” were coded as “incorrectly answered.” We created an overall MA knowledge score by calculating each respondent’s proportion of total correct responses (out of six). See Appendix A for survey questions.

Support for pharmacy dispensing of mifepristone: We measured support by asking “How supportive are you, personally, of pharmacy dispensing of mifepristone?” with a four-point Likert scale response option: very supportive, somewhat supportive, somewhat unsupportive, or very unsupportive. For the analysis, we created a dichotomous support variable (“very supportive” vs. “somewhat supportive,” “somewhat unsupportive,” and “very unsupportive”).

Additional measures: Participants’ sociodemographic background (age, gender) and professional characteristics (position, pharmacy practice site and setting, prior MA education, and medications prescribed) were collected pre-course. In the post-course survey, we collected data on respondents’ perspectives (perceived difficulty, benefits, and challenges of dispensing mifepristone), as well as training and support needs surrounding pharmacist MA dispensing, which included multiple-choice response options from which respondents could select “all that apply”.

### 2.3. Statistical Analyses

We evaluated the internal consistency of the six knowledge items, considering a Cronbach’s alpha coefficient greater than 0.70 as an acceptable threshold for using the items as a composite score [17].

To assess changes in knowledge and support from pre-course to post-course, we conducted generalized estimating equation (GEE) logistic or Poisson regression analyses with time period as the independent variable (pre-course and post-course) and each individual knowledge indicator, total number of questions correct (overall knowledge score), and support for pharmacy dispensing of mifepristone as the outcome variables. All models accounted for clustering by session and individual.

In multivariable models, we adjusted for age, gender, position, prior MA education, and pharmacy practice setting since we hypothesized these variables could influence MA knowledge or support. Due to high missingness (greater than 5%) for age, gender, and practice setting, we included “missing” as a category for these variables to retain observations in multivariable regressions. All analyses were conducted in Stata 17.0 [18].

### 2.4. Sensitivity Analyses

We conducted three sensitivity analyses to assess potential biases in our results (see Appendix A). In order to address potential selection bias that may have been introduced by attrition, we compared participant demographic characteristics (age, gender, position, pharmacy practice setting, and prior MA education) and baseline MA knowledge and support for pharmacist dispensing of MA between those in our analytic sample (completed both the pre- and post-course survey) and those who were excluded because they only completed the pre-course survey.

We also reran our main multivariable analysis restricted to only those 94 individuals who responded to all relevant sociodemographic items. Lastly, to assess differences by session, we ran a multivariable regression that included an interaction term between time and session (time × session) that combined sessions 2 and 3, given that session 2 was too small (*n* = 8) to assess independently.

## 3. Results

In total, 279 participants enrolled in the CE course and completed 277 pre-course surveys and 152 post-course surveys. We included participants who took both the pre-course and post-course surveys for the session they attended (*n* = 150); participants who took only the pre-course or post-course survey for a given session were excluded (*n* = 129). If participants took the CE multiple times and completed the pre-course and post-course surveys each time, we kept their responses for the earliest session they attended to have a more accurate measure of their pre-course knowledge (*n* = 2).

Our final analytic sample included 148 respondents: 102 (69%) from session 1, 8 (5%) from session 2, and 38 (26%) from session 3. Table 1 includes the participant sociodemographic characteristics and pharmacist practices, collected in the pre-course survey, and pharmacist perspectives from the post-course survey. Around 30% had previously received education on MA as part of their pharmacist training, and nearly 40% had ever filled a prescription for misoprostol (Table 1). Participants resided in 30 different states, with the largest proportion from California (39%).

In post-course findings, the most commonly reported possible benefits of dispensing mifepristone were improving access for patients, streamlining delivery of MA, and expanding the pharmacist’s role. The most commonly named possible challenges included pharmacies potentially refusing to stock the medications, protests or negative attention, and pharmacists potentially refusing to dispense mifepristone (Table 1).

Overall, 60% thought dispensing mifepristone would be very easy or somewhat easy to implement after completing the CE course, and 75% said they would dispense mifepristone if they were allowed to do so (Table 2).

The Cronbach’s alpha reliability coefficient was 0.76, which we considered acceptable for combining the six items into a composite score variable. In Table 2, we also show unadjusted and adjusted pre-course and post-course changes in respondents’ knowledge, for both individual knowledge items and overall knowledge scores. Unadjusted and adjusted analyses demonstrated a significant increase in the proportion of correct responses for each individual knowledge indicator, as well as for overall knowledge scores, which increased 65% from pre-test to post-test [adjusted incidence rate ratio (aIRR): 1.65, 95% CI: 1.52–1.78] (Table 2).

Table 2 also displays changes in support from pre-course to post-course. Unadjusted and adjusted analyses demonstrated the odds of responding “very supportive” were higher after taking the CE as compared to before the course [adjusted odds ratio (aOR): 2.18, 95% CI: 1.47–3.25].

The results from our sensitivity analyses are included in Appendix A. In analyses comparing the analytic sample (completed both pre- and post-course surveys) to those excluded because they only completed the pre-course survey, we found no statistically significant differences in participant sociodemographic characteristics or in MA support and knowledge (Table A1).

Table A2 includes results of the multivariable regressions from our main analysis with only observations that had no missingness on relevant sociodemographic variables. As compared to the results of our main analyses comparing changes in MA knowledge and support for MA dispensing from before to after the course, our sensitivity analysis restricted only to those with complete covariate data were similar in magnitude, direction, and significance (*p* < 0.05) across all outcome variables. The multivariable regression analysis assessing whether there were any differences in outcomes by session found no significant differences in any outcome variables, as indicated by the interaction term between time and session not being statistically significant (*p* > 0.05) (Table A3).

## 4. Discussion

Study participants’ knowledge related to MA increased significantly after completing the CE course, indicating that such a course could be an effective tool for educating pharmacists on mifepristone dispensing. Our findings are similar to a prior study measuring pharmacist knowledge before and after in-person training on MA provided in the context of a clinical trial [15]. In both studies, participants demonstrated an improved understanding of the MA regimen and policies, as well as an increase in support for pharmacist dispensing of mifepristone.

Among our study participants, 75% stated that they would dispense mifepristone if it was allowed, and support for pharmacy dispensing of mifepristone increased after the training. Under the current mifepristone REMS, certified pharmacies are able to dispense mifepristone and misoprostol with a prescription from a registered prescriber [1]. While pharmacist readiness to dispense mifepristone remains one of many barriers to increasing MA care options, qualitative findings demonstrate that pharmacists would feel more confident dispensing mifepristone if offered basic training, including online continuing education courses [19]. These types of additional resources could ensure pharmacists have the necessary tools and knowledge to offer patients quality care, particularly when medication abortion is not commonly covered in standard pharmacy education; over half of our respondents reported never having previously received education on MA.

Strong correlations exist between knowledge, experience, and confidence, highlighting the positive impacts of continuing education. Pharmacists already have various opportunities for online continuing education to expand their clinical knowledge and increase quality of patient care. A study performed in Alberta, Canada, showed that certificate courses can enhance disease management skills and expand career opportunities for pharmacists [20]. Birth Control Pharmacist, an organization dedicated to supporting pharmacists in providing reproductive health care, offers clinical resources to pharmacists, including education and training programs, policy updates, implementation tools, a digital community of practice, and research findings [21]. Specialized training programs help pharmacists stay up to date with treatment guidelines, improve patient outcomes and access to this care, and take on more advanced roles with confidence in their knowledge. The existence of such programs demonstrates the feasibility of offering additional resources and learning opportunities for pharmacists interested in dispensing medication abortion.

The most commonly reported benefit of dispensing mifepristone that respondents named was improving access for patients. Studies that have trained pharmacists to dispense or provide medication abortion have found high feasibility, satisfaction, safety, and effectiveness of the model [22,23]. Pharmacies that offer mail-order dispensing of MA have improved patient access by facilitating telemedicine provision of MA, decreasing patient travel time and offering more options for care [24,25,26]. Examples of the pharmacy-dispensing model of MA implemented in other countries, like Australia and Canada, demonstrate that pharmacists can facilitate greater access to abortion care for patients [27,28]. While many of our participants were from California, our study findings reinforce the notion that pharmacist-specific MA training and education can support implementation of MA care in U.S. pharmacies to improve patient access to MA, particularly in settings that are supportive of abortion rights [29,30,31].

We acknowledge several study limitations. While we saw a significant increase in knowledge scores, we administered the follow-up survey immediately after taking the course. We are therefore unable to measure if knowledge was retained over time or incorporated into practice. Additionally, the pre- and post-course knowledge questions were identical. Participants may have been primed to pay attention to course material that was relevant to these questions, potentially leading to an inflated increase in knowledge score. Furthermore, we lacked a comparison group which would have strengthened our ability to attribute the improvements in MA knowledge to the course itself. While attrition in our study may raise concerns of potential selection bias, the lack of baseline differences in participant sociodemographic characteristics, MA knowledge indicators, and support for pharmacist dispensing of MA between those in our analytic sample and those excluded because they were lost to follow-up mitigates some of these concerns. However, it is possible that those included differed from those excluded on unmeasured factors. Lastly, given the lack of existing validated measures of pharmacists’ knowledge of MA, we administered these six knowledge indicators based on prior research [15]. We were able to establish good internal consistency reliability and content validity of our items by developing and pilot-testing them with clinicians and pharmacists experienced in MA provision, which helped to ensure that these items accurately represented the most relevant MA knowledge concepts. However, future research should conduct more formal scale construction and psychometric evaluation of MA knowledge items, which would include testing a larger pool of items, conducting cognitive testing to ensure item comprehension, and further assessing their construct validity and correlation with pharmacist-dispensing practices [32,33,34].

Despite these limitations, our sensitivity analyses indicated our findings were reliable and robust and demonstrated improvement in MA knowledge, indicating that the CE course was an effective tool to educate participants on MA [16]. Further research should focus on measuring retention of MA knowledge from the CE course over time and how this impacts pharmacist provision of and patient access to mifepristone.

## 5. Conclusions

With patients facing greater restrictions on abortion access, it is important to prioritize efforts to expand pharmacist dispensing of mifepristone. By incorporating MA into continuing education opportunities for pharmacists, we can increase knowledge and confidence among pharmacies to provide this essential service and improve access for patients. This, in turn, can lead to more pharmacies seeking certification and more pharmacists being sufficiently equipped and willing to dispense mifepristone.

## Figures and Tables

**Table 1:** Respondent characteristics, dispensing history, and perspectives before and after taking a medication abortion continuing education module, 2020–2024 (*N* = 148).

	Pre-Course
Sociodemographic Factors and Dispensing History	*n* (%)
Age	
18–24	12 (8.1%)
25–34	38 (25.7%)
35–44	20 (13.5%)
45–54	15 (10.1%)
55+	30 (20.3%)
Missing	33 (22.3%)
Gender	
Female	101 (68.2%)
Male	35 (23.7%)
Different gender identity	2 (1.4%)
Missing	10 (6.8%)
Position	
Pharmacist	98 (66.2%)
Pharmacy student	22 (14.9%)
Other	21 (14.2%)
Missing	7 (4.7%)
Pharmacy practice setting	
Rural	21 (14.2%)
Urban	56 (37.8%)
Suburban	41 (27.7%)
Missing	30 (20.3%)
Primary practice site *	
Community pharmacy, chain	30 (20.3%)
Hospital or health-system	20 (13.5%)
Community pharmacy, independent	19 (12.8%)
Outpatient pharmacy at clinic or hospital	14 (9.5%)
Student health center pharmacy	11 (7.4%)
Ambulatory clinic	11 (7.4%)
Academia	13 (8.8%)
Other	13 (8.8%)
N/A, student	10 (6.8%)
Missing	7 (4.7%)
Medication abortion covered in education	
Pharmacy school only	38 (25.7%)
Post-grad training only	7 (4.7%)
Both pharmacy school and post-grad	13 (8.8%)
None of the above	82 (55.4%)
Missing	8 (5.4%)
Currently prescribe *	
Immunizations	62 (41.9%)
Emergency contraception	53 (35.8%)
Hormonal contraception	50 (33.8%)
Other	7 (4.7%)
None of the above	45 (30.4%)
Missing	9 (6.1%)
Ever filled prescription for misoprostol	
Yes	59 (39.9%)
No	79 (53.4%)
Missing	10 (6.8%)
	Post-course
Perspectives on continuing education course and mifepristone dispensing	*n* (%)
Difficulty or ease of mifepristone dispensing	
Very easy	32 (21.6%)
Somewhat easy	57 (38.5%)
Somewhat difficult	39 (26.4%)
Very difficult	8 (5.4%)
Missing	12 (8.1%)
Perceived benefits of dispensing mifepristone *	
Improve access	127 (85.8%)
Streamline delivery	112 (75.7%)
Expand pharmacist’s role	112 (75.7%)
Other	2 (1.4%)
N/A, no benefits	3 (2.0%)
Missing	11 (7.4%)
Perceived challenges of dispensing mifepristone *	
Pharmacies may refuse to stock	80 (54.1%)
Protests or negative attention	64 (43.2%)
Pharmacists may refuse to dispense	60 (40.5%)
Pharmacists not familiar with medication	55 (37.2%)
Unable to answer patient questions	45 (30.4%)
Unable to determine safe use	45 (30.4%)
Other	4 (2.7%)
N/A, no challenges	19 (12.8%)
Missing	13 (8.8%)
Which topics do you feel need additional training, after completing the continuing education course? *	
Abortion counseling	68 (46.0%)
Drug interactions	39 (26.4%)
Side effects and adverse events	30 (20.3%)
Mechanism of action	29 (19.6%)
Regimen details	23 (15.5%)
Efficacy of regimen	19 (12.8%)
Other	2 (1.4%)
None of the above	27 (18.2%)
Missing	12 (8.1%)
Which of the following tools would help you dispense mifepristone and misoprostol in a community pharmacy setting? *	
Patient education materials	105 (71.0%)
Patient counseling guide	103 (69.6%)
Clinical guidelines	70 (47.3%)
Counseling videos	61 (41.2%)
Educational programs	61 (41.2%)
Pharmacy policies/procedures	61 (41.2%)
Primary literature	40 (27.0%)
Other	2 (1.4%)
Missing	12 (8.1%)
If allowed, would you dispense mifepristone?	
Yes	111 (75.0%)
No (refuse)	8 (5.4%)
Not sure	9 (6.1%)
N/A, does not practice at a pharmacy	6 (4.1%)
Missing	14 (9.5%)

* Select all that apply—multiple options may have been selected.

**Table 2:** Bivariable and multivariable results of changes in respondent knowledge of and support for pharmacy dispensing of mifepristone before and after taking a medication abortion continuing education course.

Knowledge Indicators	Pre-Course	Post-Course	Bivariable Analyses (*N* = 148) *	Multivariable Analyses (*N* = 140) **
	*n* (%)	*n* (%)	Odds Ratio (OR) (95% CI)	Adjusted Odds Ratio (aOR) (95% CI)
Mifepristone mechanism of action	73 (49.3%)	128 (86.5%)	7.11 (4.22–12.00)	8.40 (4.70–14.96)
Misoprostol mechanism of action	119 (80.4%)	139 (93.9%)	3.78 (1.81–7.87)	4.35 (1.97–9.55)
Misoprostol dosage and administration	45 (30.4%)	128 (86.5%)	15.37 (9.00–26.23)	23.01 (11.89–44.55)
Common side effects	86 (58.1%)	131 (88.5%)	5.68 (3.29–9.79)	6.29 (3.51–11.29)
Postabortion return to fertility	91 (61.5%)	130 (87.8%)	4.69 (2.91–7.55)	6.42 (3.64–11.34)
Gestational duration limit in Food and Drug Administration (FDA) labeling	65 (43.9%)	133 (89.9%)	12.16 (7.02–21.07)	17.01 (8.79–32.91)
Overall Knowledge Score (number correct out of 6)	*n* (%)	*n* (%)	Incidence Rate Ratio (IRR) (95% CI)	Adjusted Incidence Rate Ratio (aIRR) (95% CI)
0	5 (3.4%)	0 (0.0%)	1.65 (1.53–1.78)	1.65 (1.52–1.78)
1	18 (12.2%)	5 (3.4%)		
2	29 (19.6%)	5 (3.4%)		
3	32 (21.6%)	5 (3.4%)		
4	30 (20.3%)	7 (4.7%)		
5	17 (11.5%)	25 (16.9%)		
6	17 (11.5%)	101 (68.2%)		
Support for Pharmacy Dispensing of Mifepristone	*n* (%)	*n* (%)	OR (95% CI)	aOR (95% CI)
Very supportive	91 (61.5%)	108 (73.0%)	2.07 (1.42–3.04)	2.18 (1.47–3.25)
Somewhat supportive	30 (20.3%)	17 (11.5%)		
Somewhat unsupportive	11 (7.4%)	3 (2.0%)		
Very unsupportive	10 (6.8%)	10 (6.8%)		
Missing	6 (4.1%)	10 (6.8%)		

All regression analyses were statistically significant at *p* < 0.001. * Adjusted for clustering by session and individual. ** Adjusted for clustering by session and individual, as well as age, gender, position, prior medication abortion education, and pharmacy practice setting.

## Data Availability

The original contributions presented in this study are included in the article. Further inquiries can be directed to the corresponding author.

## Appendix A

Medication abortion knowledge questions from surveys of participants of medication abortion continuing pharmacy education program.

Correct answers are check-marked.

Please answer the following questions about medication abortion to the best of your knowledge. If you do not know the answer, feel free to select “don’t know.”

1. **Mifepristone interferes with a pregnancy by competitively blocking the estrogen receptor.**
  ☐   True
  ⊠   False
  ☐   Don’t know
2. **Misoprostol causes cervical dilation and uterine contractions to empty the uterus.**
  ⊠   True
  ☐   False
  ☐   Don’t know
3. **Mifepristone 200 mg is FDA-approved for use up to how many days after the last menstrual period?**
  ☐   49 days (7 weeks)
  ☐   56 days (8 weeks)
  ☐   63 days (9 weeks)
  ⊠   70 days (10 weeks)
  ☐   Don’t know
4. **Because return to fertility is usually delayed by at least 6 weeks, patients should postpone the initiation of contraception after medication abortion.**
  ☐   True
  ⊠   False
  ☐   Don’t know
5. **Following mifepristone, how should patients be advised to take the misoprostol dose?**
  ☐   Misoprostol 200 mcg orally 12–24 h later
  ☐   Misoprostol 400 mcg buccally 12–24 h later
  ☐   Misoprostol 600 mcg orally 24–48 h later
  ⊠   Misoprostol 800 mcg buccally 24–48 h later
  ☐   Don’t know
6. **Which of the following is NOT a common side effect of the mifepristone-misoprostol medication abortion regimen?**
  ☐   Bleeding
  ☐   Cramping
  ☐   Nausea/vomiting
  ☐   Fever/chills
  ⊠   Thrombocytopenia
  ☐   Don’t know

Sensitivity analyses to assess potential biases.

**Table A1:** Pre-course responses to sociodemographic, support, and knowledge items, with bivariate regression analyses assessing differences between those excluded because they only completed the pre-course survey (*n* = 127) and those who completed both pre- and post-course surveys (*n* = 148).

Pre-Course Support or Knowledge Item	Excluded Sample (Pre-Course Only) (*n* = 127)	Analytic Sample (Pre- and Post-Course) (*n* = 148)		
	*n* (%)	*n* (%)	OR (95% CI)	*p*-value
Age				
18–24	6 (4.7%)	12 (8.1%)	Ref.	-
25–34	31 (24.4%)	38 (25.7%)	0.61 (0.21–1.83)	0.379
35–44	20 (15.8%)	20 (13.5%)	0.50 (0.16–1.60)	0.243
45–54	12 (9.5%)	15 (10.1%)	0.63 (0.18–2.17)	0.459
55+	20 (15.8%)	30 (20.3%)	0.75 (0.24–2.33)	0.619
Missing	38 (29.9%)	33 (22.3%)	-	-
Gender				
Male	24 (18.9%)	35 (23.7%)	Ref.	-
Female or “other” gender identity	97 (76.4%)	103 (69.6%)	0.73 (0.40–1.31)	0.292
Missing	6 (4.7%)	10 (6.8%)	-	-
Position				
Pharmacist	89 (70.1%)	98 (66.2%)	Ref.	-
Pharmacy student	12 (9.5%)	22 (14.9%)	1.66 (0.78–3.56)	0.189
Other	21 (16.5%)	21 (14.2%)	1.00 (0.51–1.99)	0.991
Missing	5 (3.9%)	7 (4.7%)	-	-
Pharmacy practice setting				
Rural	20 (15.8%)	21 (14.2%)	Ref.	-
Urban	45 (35.4%)	56 (37.8%)	1.19 (0.57–2.46)	0.648
Suburban	40 (31.5%)	41 (27.7%)	0.98 (0.46–2.07)	0.950
Missing	22 (17.3%)	30 (20.3%)	-	-
Medication abortion covered in education				
Both pharmacy school and post-grad	6 (4.7%)	13 (8.8%)	Ref.	-
Pharmacy school only	26 (20.5%)	38 (25.7%)	0.67 (0.23–2.01)	0.479
Post-grad training only	12 (9.5%)	7 (4.7%)	0.27 (0.07–1.03)	0.056
None of the above	74 (58.3%)	82 (55.4%)	0.51 (0.18–1.42)	0.197
Missing	9 (7.1%)	8 (5.4%)	-	-
Support	*n* (%)	*n* (%)	OR (95% CI)	*p*-value
Very supportive	85 (66.9%)	91 (64.1%)	0.88 (0.53–1.46)	0.625
Somewhat supportive	25 (19.7%)	30 (21.1%)		
Somewhat unsupportive	10 (7.9%)	11 (7.8%)		
Very unsupportive	7 (5.5%)	10 (7.0%)		
Knowledge indicators	*n* (%)	*n* (%)	OR (95% CI)	*p*-value
Mifepristone mechanism of action	62 (48.8%)	73 (49.3%)	1.02 (0.63–1.64)	0.934
Misoprostol mechanism of action	102 (80.3%)	119 (80.4%)	1.01 (0.55–1.83)	0.985
Gestational duration limit in FDA labeling	59 (46.5%)	65 (43.9%)	0.90 (0.56–1.45)	0.674
Postabortion return to fertility	78 (61.4%)	91 (61.5%)	1.00 (0.62–1.63)	0.991
Misoprostol dosage and administration	38 (29.9%)	45 (30.4%)	1.02 (0.61–1.72)	0.931
Common side effects	83 (65.4%)	86 (58.1%)	0.74 (0.45–1.20)	0.220
Overall knowledge score (number correct)	*n* (%)	*n* (%)	IRR (95% CI)	*p*-value
0	8 (6.3%)	5 (3.4%)	Ref.	-
1	11 (8.7%)	18 (12.2%)	2.62 (0.68–10.08)	0.162
2	26 (20.5%)	29 (19.6%)	1.78 (0.51–6.16)	0.359
3	21 (16.5%)	32 (21.6%)	2.44 (0.70–8.49)	0.162
4	26 (20.5%)	30 (20.3%)	1.85 (0.54–6.36)	0.331
5	18 (14.2%)	17 (11.5%)	1.51 (0.41–5.55)	0.534
6	17 (13.4%)	17 (11.5%)	1.60 (0.43–5.91)	0.481

OR: Odds ratio; CI: confidence interval; IRR: incident rate ratio.

**Table A2:** Multivariable regression analyses of changes in knowledge of and support for pharmacy dispensing of mifepristone from pre- to post-course, among restricted sample of participants who responded to all relevant sociodemographic items (i.e., no missingness in covariates) (*n* = 94).

Outcome Variable	aOR (95% CI)	*p*-Value
Support (very supportive)	1.80 (1.15–2.80)	0.010
Knowledge items		
Mifepristone mechanism of action	6.63 (3.24–13.59)	<0.001
Misoprostol mechanism of action	5.37 (1.88–15.40)	0.002
Gestational duration limit in FDA label	15.57 (6.76–35.88)	<0.001
Postabortion return to fertility	5.29 (2.74–10.22)	<0.001
Misoprostol dosage and administration	16.80 (7.64–36.96)	<0.001
Common side effects	5.29 (2.62–10.68)	<0.001
	aIRR (95% CI)	*p*-value
Overall knowledge score	1.58 (1.44–1.72)	<0.001

All models adjusted for clustering by session and individual, as well as age, gender, position, prior MA education, and pharmacy practice setting. aOR: adjusted Odds Ratio; aIRR: adjusted Incident Rate Ratio.

**Table A3:** Multivariable regression analysis assessing differences between session mode (*n* = 140).

Outcome Variable	aOR (95% CI)	*p*-Value
Mifepristone mechanism of action		
Time		
Pre-course	Ref.	-
Post-course	8.62 (4.44, 16.72)	<0.001
Session		
Session 1	Ref.	-
Sessions 2 and 3	1.40 (0.64, 3.06)	0.395
Time × session interaction term	0.79 (0.21, 2.87)	0.715
Misoprostol mechanism of action		
Time		
Pre-course	Ref.	-
Post-course	3.44 (1.48, 7.99)	0.004
Session		
Session 1	Ref.	-
Sessions 2 and 3	1.42 (0.54, 3.73)	0.480
Time × session interaction term	3.03 (0.30, 30.37)	0.346
Misoprostol dosage and administration		
Time		
Pre-course	Ref.	-
Post-course	26.58 (11.92, 59.27)	<0.001
Session		
Session 1	Ref.	-
Sessions 2 and 3	1.77 (0.79, 3.94)	0.166
Time × session interaction term	0.58 (0.16, 2.16)	0.418
Common side effects		
Time		
Pre-course	Ref.	-
Post-course	5.85 (2.94, 11.63)	<0.001
Session		
Session 1	Ref.	-
Sessions 2 and 3	1.56 (0.69, 3.54)	0.287
Time × session interaction term	1.28 (0.34, 4.79)	0.713
Postabortion return to fertility		
Time		
Pre-course	Ref.	-
Post-course	5.75 (3.00, 11.01)	<0.001
Session		
Session 1	Ref.	-
Sessions 2 and 3	1.27 (0.48, 3.40)	0.630
Time × session interaction term	1.65 (0.37, 7.43)	0.516
Gestational duration limit in FDA labeling		
Time		
Pre-course	Ref.	-
Post-course	17.32 (7.77, 38.62)	<0.001
Session		
Session 1	Ref.	-
Sessions 2 and 3	0.54 (0.23, 1.29)	0.165
Time × session interaction term	0.91 (0.26, 3.23)	0.890
Support for pharmacy dispensing of mifepristone		
Time		
Pre-course	Ref.	-
Post-course	2.03 (1.24, 3.33)	0.005
Session		
Session 1	Ref.	-
Sessions 2 and 3	0.52 (0.23, 1.18)	0.119
Time × session interaction term	1.21 (0.53, 2.78)	0.650
	aIRR (95% CI)	*p*-value
Overall knowledge score		
Time		
Pre-course	Ref.	-
Post-course	1.69 (1.54, 1.87)	<0.001
Session		
Session 1	Ref.	-
Sessions 2 and 3	1.10 (0.94, 1.28)	0.239
Time × session interaction term	0.92 (0.78, 1.07)	0.268

All models adjusted for clustering by session and individual, as well as age, gender, position, prior MA education, and pharmacy practice setting. aOR: adjusted Odds Ratio; aIRR: adjusted Incident Rate Ratio.
